# Gene and MicroRNA Expression Responses to Exercise; Relationship with Insulin Sensitivity

**DOI:** 10.1371/journal.pone.0127089

**Published:** 2015-05-18

**Authors:** Carrie S. McLean, Clinton Mielke, Jeanine M. Cordova, Paul R. Langlais, Benjamin Bowen, Danielle Miranda, Dawn K. Coletta, Lawrence J. Mandarino

**Affiliations:** 1 School for the Science of Health Care Delivery, Arizona State University, Tempe, Arizona, United States of America; 2 School of Life Sciences, Arizona State University, Tempe, Arizona, United States of America; 3 Mayo Clinic in Arizona, Scottsdale, Arizona, United States of America; INSERM/UMR 1048, FRANCE

## Abstract

**Background:**

Healthy individuals on the lower end of the insulin sensitivity spectrum also have a reduced gene expression response to exercise for specific genes. The goal of this study was to determine the relationship between insulin sensitivity and exercise-induced gene expression in an unbiased, global manner.

**Methods and Findings:**

Euglycemic clamps were used to measure insulin sensitivity and muscle biopsies were done at rest and 30 minutes after a single acute exercise bout in 14 healthy participants. Changes in mRNA expression were assessed using microarrays, and miRNA analysis was performed in a subset of 6 of the participants using sequencing techniques. Following exercise, 215 mRNAs were changed at the probe level (Bonferroni-corrected P<0.00000115). Pathway and Gene Ontology analysis showed enrichment in MAP kinase signaling, transcriptional regulation and DNA binding. Changes in several transcription factor mRNAs were correlated with insulin sensitivity, including MYC, r=0.71; SNF1LK, r=0.69; and ATF3, r= 0.61 (5 corrected for false discovery rate). Enrichment in the 5’-UTRs of exercise-responsive genes suggested regulation by common transcription factors, especially EGR1. miRNA species of interest that changed after exercise included miR-378, which is located in an intron of the PPARGC1B gene.

**Conclusions:**

These results indicate that transcription factor gene expression responses to exercise depend highly on insulin sensitivity in healthy people. The overall pattern suggests a coordinated cycle by which exercise and insulin sensitivity regulate gene expression in muscle.

## Introduction

The global gene expression response of skeletal muscle to acute exercise has been characterized recently in healthy men [1]. In the immediate post-exercise period, many genes are increased in expression, and among these are transcription factors (NR4A, EGR1, JUNB, FOS), angiogenic factors such as CYR61, proteins involved in extracellular matrix turnover such as ADAMTS4, and genes in the MAP kinase signaling pathway. The enrichment of transcription factors in exercise early-responsive genes suggests that there is a coordinated transcriptional response that regulates gene expression responses to acute exercise and exercise training, including increases in expression of genes involved in mitochondrial function and aerobic metabolism, which are linked to insulin action [2].

Skeletal muscle contraction and insulin action are inter-twined [3, 4]. Acute exercise and exercise training have a variety of effects on gene expression, ranging from effects on GLUT4 expression to mitochondrial biogenesis and adaptations in structural proteins [5–15]. In addition to its effects on aerobic capacity and performance, exercise improves insulin sensitivity in skeletal muscle. However, it has become clear that greater insulin sensitivity itself also influences the acute gene expression response of skeletal muscle to exercise [6], potentially leading to a feed-forward virtuous cycle.

There is a broad range of insulin sensitivity in skeletal muscle in healthy humans [2]. We have shown that even among healthy, nondiabetic individuals, those on the lower end of the distribution of insulin action have lower gene expression responses to exercise [6]. This pattern of expression differences suggests that there may be different transcription factor responses to exercise that are related to insulin sensitivity in healthy individuals. Previous studies have shown that PGC-1α mRNA and protein responses to exercise may in part be responsible for some of these differences [6]. To date, however, there has been no global, unbiased analysis that has identified an array of exercise-induced transcription factors and other genes that might be related to insulin sensitivity. Therefore, the primary purpose of this study was to determine whether in healthy individuals there is insulin sensitivity-based variation in exercise-induced early response of skeletal muscle genes, particularly those coding for transcription factors.

It also has become evident that not only does gene expression in muscle change after acute exercise, but the expression of microRNAs (miRNAs) also can be affected by endurance exercise [13] resistance exercise [16], aging [17], and plays a role in muscle plasticity [18]. miRNAs are small, 22–25 nt RNA species that are widespread throughout the genome. They reside in introns of genes or in other noncoding regions and act by binding to the 3’UTR of messages to decrease translation or mRNA stability [19]. When miRNAs are present within introns, they often participate in regulation of the pathways involving the “parent” gene. Although miRNAs generally reduce abundance of proteins coded by the mRNAs with which they interact, they also can increase protein and mRNA abundance when they target inhibitors of transcription. Several miRNAs respond to exercise [13, 16], although no unbiased, global analysis has been done, and there is very little known about this or whether miRNA expression after exercise might be related to insulin sensitivity. Therefore, the second purpose of this study was to characterize the global miRNA response to acute exercise in muscle from healthy volunteers with a wide range of insulin sensitivity.

## Methods

### Participants

**Fig 1:**
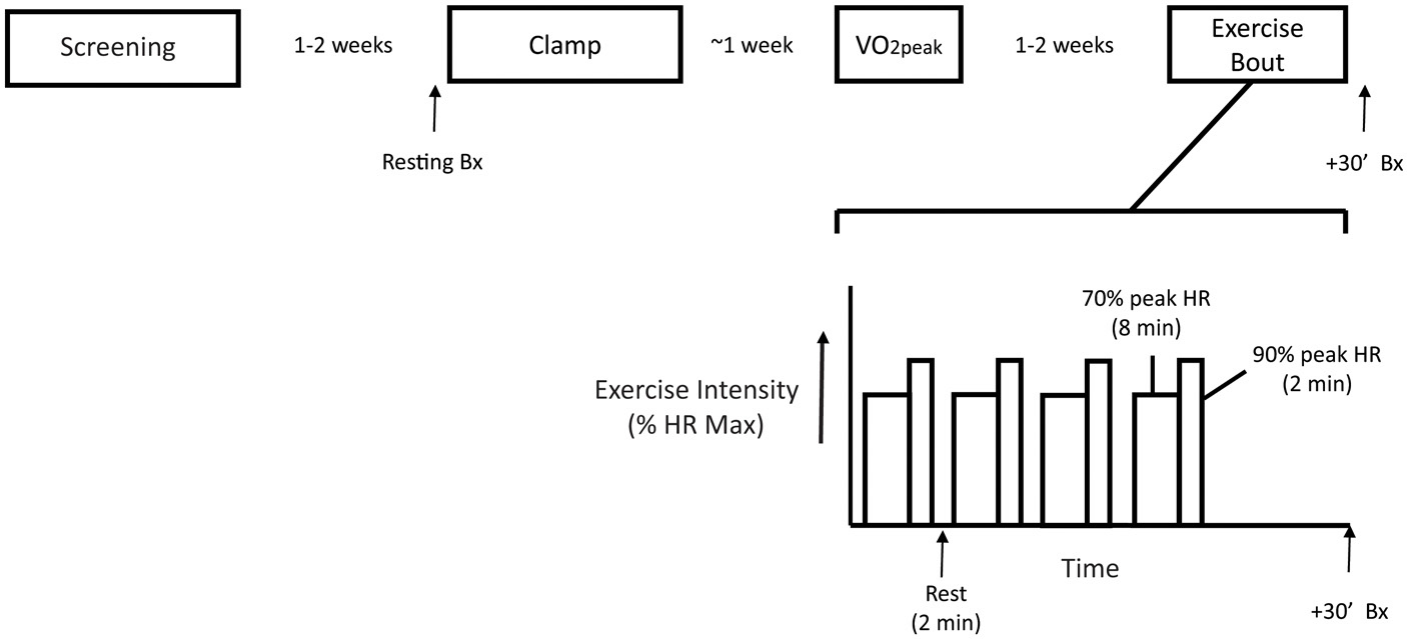
Overall design of the study. The overall sequence of study days is shown above an expanded view of the acute exercise bout. During the acute exercise bout, subjects exercise for a total of 48 minutes, consisting of 4 sets of exercise, each set consisting of 8 minutes at 70% HR max, 2 minutes at 90% HR max, and 2 minutes of rest. A biopsy of the *vastus lateralis* muscle was taken at 30 minutes after completing the four sets of exercise. Bx, Biopsy; VO_2_, rate of oxygen consumption; HR, Heart Rate.

Fourteen normoglycemic volunteers took part in this study, which was approved by the Institutional Review Board of Arizona State University. All studies were conducted at the Clinical Research Unit at ASU. Informed, written consent was obtained from all subjects. All of the volunteers were sedentary, and no one reported having a change in body weight for at least 6 months before participating in this study. Subjects were instructed not to exercise for 48 hours before study and to maintain their usual diet. All subjects were sedentary and did not report a family history of type 2 diabetes. A medical history, physical examination, 12-lead electrocardiogram, and a complete chemistry panel were obtained, and a 75-g oral glucose tolerance test was performed using American Diabetes Association criteria. No one was taking any medication known to affect glucose metabolism. The design of the study is shown in Fig 1.

### Procedures

#### Peak aerobic capacity

Peak aerobic capacity (VO_2peak_) was determined as previously described, with continuous heart rate monitoring [5]. Exercise was started at a workload of 40 W and increased by 10 W/min until perceived exhaustion or a respiratory quotient of 1.10 was reached.

#### Muscle biopsy and hyperinsulinemic euglycemic clamp

Euglycemic clamps and muscle biopsies were performed as described [20]. On a day separated from exercise tests by at least one week, and after a 10-h overnight fast, a percutaneous biopsy of the *vastus lateralis* muscle was obtained with a Bergstrom cannula under local anesthesia one hour before the start of insulin infusion. This biopsy served as the resting, non-exercised control. An insulin infusion rate of 80 mU·m^-2^ ·min^-1^ was used. The rate of glucose infusion required to maintain euglycemia during the last 30 minutes of the insulin infusion was taken as an estimate of insulin-stimulated glucose disposal, since this insulin infusion rate effectively suppresses endogenous glucose production [20].

#### Exercise bout with muscle biopsies

All subjects underwent a single bout of aerobic exercise (Fig 1), which was conducted on a separate day after determination of VO_2peak_, as described previously [6], and at least 1 week after the euglycemic hyperinsulinemic clamp. Subjects reported to the Clinical Research Unit at about 7 AM after fasting overnight and exercised on a stationary bicycle. Immediately after completing exercise, the subject had a biopsy of the *vastus lateralis* muscle within 30 min of the end of exercise. The total length of the exercise bout was 48 minutes, consisting of 4 sets, each composed of 8 minutes of cycling at 70% peak heart rate, 2 minutes at 90% peak, and 2 minutes rest.

#### RNA isolation, microarray processing and quantitative real time PCR analysis

Muscle RNA was isolated as described [21]. Microarray analysis using the 4x44K Whole Human Genome Microarrays (Agilent Technologies, Palo Alto, CA) was performed as previously described [22]. Quantitative real time PCR analyses for selected mRNAs were performed as described [6]. Primers used for these analyses are given in S1 Table.

#### miRNA analysis

For miRNA analysis, RNA was isolated from frozen muscle using the miRNeasy kit (Qiagen, Valencia, CA). Small RNA libraries were prepared according to manufacturer’s instructions for the NEBNext Multiplex Small RNA Kit (New England Biolabs; Ipswich, MA). miRNA sequencing was performed on Illumina HiSeq 2000 sequencers (Illumina, San Diego, CA) using Illumina’s standard protocol using the Illumina cBot and cBot Paired end cluster kit version 3. A total of 2227 miRNAs were sequenced; 16.0% of these probes were present in all specimens, 26.4% were present in greater than one half of subjects. In total, miRNA sequences were positively called for 27.3% of all 2227 possible sequences. The raw data files, metadata and matrix table were deposited in the Gene Expression Omnibus (GEO) (GSE66334).

#### Bioinformatics/statistics

Gene expression values were compared at the probe level. Expression values for each probe on the array were log transformed. Pre and post exercise gene expression values in all subjects were compared by paired t-test, and P values were adjusted using the Bonferroni correction (P < 0.00000115). To test the hypothesis that insulin sensitivity is correlated with exercise-induced changes in gene expression, probe signals for mRNAs that significantly responded to exercise (stringent Bonferroni corrected P value) were correlated with rates of insulin stimulated glucose disposal determined with the euglycemic clamps, using the Pearson product moment method (significance set at P<0.05). The raw data files, metadata and matrix table were deposited in the Gene Expression Omnibus (GEO) (http://www.ncbi.nlm.nih.gov/geo/query/acc.cgi?acc=GSE43219).

Analysis of the 5’ untranslated regions (UTR) of genes responsive to exercise was conducted using PScan software [23], using both the JASPAR [24] and TRANSFAC databases [25]. We used the -950 to +50 5’ UTR region of genes in PScan as the control for statistical analysis and used the stringent Bonferroni P values to determine significance of enrichment of response elements in promoters. Transcription factor binding motif enrichment concordant using both databases was considered significant. For miRNA sequencing, SCS version 1.4.8 data collection software was used. Base-calling was performed using Illumina’s RTA version 1.12.4.2. A miRNA data analysis pipeline using Casava 1.8 and Flicker 3.0 was used to generate miRNA gene list counts. miRNA target analysis was performed using the microT-CDS tool available through Diana Tools (http://diana.imis.athena-innovation.gr/DianaTools/index.php?r=site/index) [26, 27]. Database for Annotation, Visualization and Integrated Discovery (DAVID) (http://david.niaid.nih.gov) was used for all KEGG pathway and Gene Ontology (GO) analysis.

#### Analytical determinations

Plasma insulin concentrations were measured by radioimmunoassay (Diagnostic Product, Los Angeles, CA). Tracer to tracee ratio of glucose ([6,6-^2^H] glucose/glucose) was determined using selective ion monitoring on a Finnigan Trace DSQ GS/MS (Thermo Electron Corporation, Waltham, MA, USA). Total cholesterol, triglyceride and HDL were measured by Sonora Quest Laboratories, Phoenix, AZ.

## Results

### Subject characteristics, insulin sensitivity, and exercise bout

Participant characteristics are given in Table 1. A total of 14 subjects took part in the study. All subjects had normal glucose tolerance and were healthy, with a wide range of insulin sensitivity by design (5.2–13.3 mg·(kg FFM)^-1^·min^-1^). All subjects were sedentary. Peak aerobic exercise capacity (VO_2peak_) for all 14 subjects was not correlated with insulin stimulated glucose disposal (r = 0.08, P = NS), or BMI (r = -0.02, P = NS). Subjects achieved the desired 70 and 90% of maximum heart rates during each of the four sets of exercise. Characteristics of the subset of 6 (of the total of 14 subjects) who also had miRNA determinations also are given in Table 1. Exercise characteristics of the participants also are given in Table 1. Subjects achieved the desired 70 and 90% if peak heart rate values during each of the 4 sets of the exercise bout.

**Table 1:** Participant characteristics.

	Total	Median (range)	miRNA subset	Median (range)
Sex	9M/5F		5M/1F	
Age (years)	33 ± 2	33 (23–48)	33 ± 3	31 (26–48)
Body Mass Index (Kg/m^2^)	27.2 ± 0.8	26.5 (22.1–34.2)	27.2 ± 0.7	26.7 (25.5–29.9)
Body fat (%)	25.5 ± 1.3	26.2 (15–34)	22.0 ± 1.7	22.2 (15–26.2)
Fasting plasma glucose (mM)	5.00 ± 0.06	5.00 (4.5–5.4)	4.99 ± 0.05	4.94 (4.9–5.2)
HbA1c (%)	5.4 ± 0.1	5.5 (4.9–5.8)	5.4 ± 0.2	5.4 (4.9–5.8)
Cholesterol (mg/dL)	177 ± 6	176 (145–213)	171 ± 10	161 (145–206)
HDL Cholesterol (mg/dL)	50.3 ± 3.4	47.5 (33–84)	44.7 ± 1.9	44 (40–52)
LDL Cholesterol (mg/dL)	106 ± 4	107 (80–146)	101 ± 5	97 (87–117)
VLDL Cholesterol (mg/dL)	20 ± 3	16 (7–43)	25 ± 6	22 (7–43)
Triglycerides (mg/dL)	118 ± 17	96 (44–258)	148 ± 32	133 (44–258)
Fasting plasma insulin (pM)	41 ± 7	35 (11–100)	44 ± 13	33 (11–100)
Clamp plasma insulin (pM)	944 ± 58	912 (684–1434)	878 ± 81	87 (684–1068)
Insulin stimulated glucose disposal (mg·Kg FFM^-1^·min^-1^)	9.3 ± 0.8	8.9 (5.2–13.3)	9.7 ± 1.2	8.9 (6.1–13.3)
VO_2peak_ (ml·Kg FFM^-1^·min^-1^)	43.8 ± 2.1	42.8 (31.9–58.3)	43.3 ± 3.4	41.8 (34.2–58.4)
Resting Heart Rate (BPM)	65 ± 2	66 (58–76)	64 ± 2	64 (58–71)
Peak Heart Rate (BPM) actual(predicted)	184 ± 2(187 ± 2)	184 (172–199)	181 ± 2(187 ± 2)	177 (173–199)
70% Heart Rate (BPM) actual(predicted)	138 ± 2**(129 ± 2)	136 (123–159)	137 ± 4*(126 ± 3)	135 (123–152)
90% Heart Rate (BPM) actual(predicted)	162 ± 3(166 ± 2)	160 (144–179)	160 ± 3(162 ± 4)	160 (150–169)
Maximum Work (Watts)	175 ± 18	175 (100–260)	198 ± 18	200 (125–260)

Data are given as Mean ± SEM. The six volunteers in the miRNA subset are included in the total group of 14. Data are shown as Mean ± SEM. Peak heart rate was highest value achieved during the VO_2peak_ determination. Maximum predicted heart rate = 220—age.

*P<0.05,

**P < 0.01 vs. predicted value.

Kg, kilograms; mg, milligrams; dL, deciliter; pM, picomolar; FFM, fat free mass; BPM, beats per minute.

### Effects of exercise on gene expression in skeletal muscle

Changes in gene expression were assessed using RNA isolated from muscle samples taken under resting conditions and 30 minutes after the completion of exercise. In all subjects, exercise altered mRNA expression significantly for 215 probes (S2 Table, Bonferroni corrected P < 0.00000115). The majority of these probes, nearly 200, increased in expression. Prominent among the genes represented by the probes that increased in expression were transcription factors (54/215 significant probes), including MYC, JUN, FOS, ATF3, ID1-3, and CTGF. Probes for chemokines and related genes also were well represented (16/215), and included CX3CL1, CCL2, and two metallopeptidases, ADAMTS4 and ADAMTS1. Probes for genes involved in regulation of cell cycle (9/215) included GADD45B and RASSF1. Probes for genes encoding proteins involved in angiogenesis also were represented, including CYR61 and VEGFa. Although mRNA for only one gene encoding a mitochondrial protein was increased significantly (SLC25A25, mitochondrial phosphate carrier), a number of others increased but did not achieve the Bonferroni cutoff. Among these were several Complex I and ATP synthase subunits, SLC2A4, or GLUT4 (P = 0.005) and PPARGC1B (P = 0.039). Only 17 mRNAs decreased significantly by the Bonferroni criterion. Among these were isoforms 1,4,6,7, and 8 of the GIMAP family of nucleotide binding proteins with GTPase activity. David analysis of the probes that had Bonferroni significant changes after exercise in all subjects showed 4.0 and 7.7-fold enrichment (false discovery rate ≤ 5%) in MAP kinase and TGFβ signaling pathways (S3 Table). Enrichment in GO molecular function terms was significant for regulation of transcription and GTP binding in the 215 probes entered into DAVID for analysis.

### Quantitative rt-PCR assessment of gene expression

To confirm the microarray results, several mRNAs (EGR1, FOS, MYC, JUND, and CTGF) were selected for Q-rt-PCR assay of changes in gene expression induced by the exercise bout, compared with microarray results (S1 Fig). Changes in gene expression determined by Q-rt-PCR were concordant with changes in gene expression determined by microarray analysis (r = 0.86, P<0.05).

#### Analysis of 5’UTR of genes responding to exercise

To understand which transcription factors might be responsible for the exercise-induced responses of the 130 exercise-induced genes represented by the 215 probes, we analyzed the promoter regions (-950 to +50 bp) of these genes for enrichment in transcription factor response element binding motifs using PScan [23]. We used response element profiles available in both the JASPAR [24] and TRANSFAC [28] databases. Transcription factors of interest that were enriched in the promoter regions of exercise-responsive genes included SP1, KLF4, NFKB, RELA, and EGR1 (S4 Table). The correlation between insulin stimulated glucose disposal and exercise induced changes in gene expression was significant for the transcription factors NFKB1 and RELA.

### Relationships between insulin sensitivity and gene expression response to exercise

**Fig 2:**
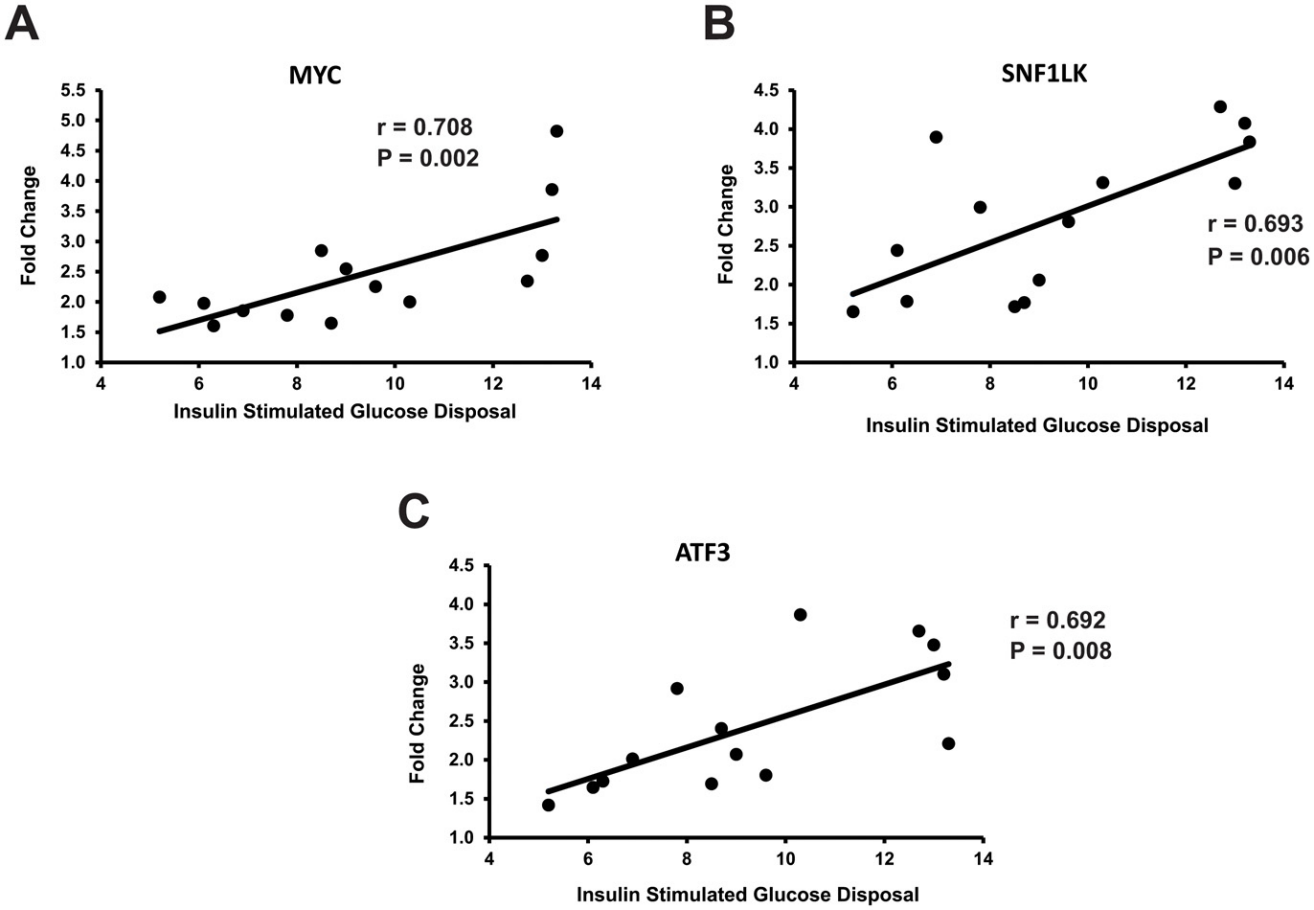
Relationships among response of genes (at the probe level) to exercise and insulin sensitivity. Shown are exercise-induced fold changes in expression for the indicated genes plotted against rates of insulin-stimulated glucose disposal during a euglycemic, hyperinsulinemic clamp. P values displayed for correlations were corrected using the Benjamini Hochberg method.

To determine the relationships between insulin sensitivity and significant gene expression changes after exercise, fold changes in Bonferroni significant gene expression were correlated with rates of insulin stimulated glucose disposal during a euglycemic clamp. The comparison was done at the probe level, using P values corrected for a false discovery rate of 0.25 using the Benjamini Hochberg method. The results for the three significant correlations are shown in Fig 2, and included MYC, ATF3, and SNF1LK.

#### Effects of exercise on miRNA expression

To quantify miRNA expression, we isolated RNA separately (see Methods) from a subset of 6 individuals who had sufficient muscle biopsy material remaining after mRNA analyses. miRNA expression was quantified using next generation sequencing techniques from biopsies taken basally and 30 minutes after completion of the exercise bout. Data, expressed as normalized reads and log_2_ (fold increase with exercise) are given in Table 2. Thirteen miRNA species were altered significantly (Bonferroni corrected P value) after exercise; all of these increased. Among these, several miRNAs in the miR-378 family, namely miR-378a-3p, -378a-5p, -378f, -378g, and -378i all increased. In general, “-3p” species of miRNAs from a stem loop sequence are more abundant than the “-5p” species, being selectively included in the RISC complex [29]. In keeping with this, miR-378a-3p was dramatically more abundant than miR-378a-5p. miR-30a-5p and miR-30d-5p also significantly increased. Only two miRNAs decreased after exercise, miR-144-5p and -144-3p, but these decreases were not statistically significant. Changes in miRNA were not correlated with insulin sensitivity. Several miRNAs were in introns of genes involved in metabolic events, including miR-378a-3p and miR-378-5p (intron 1 of PPARGC1B, which also increased after exercise) and miR-378f (within the CNR2, cannabinoid receptor 2 gene). We also searched predicted mRNA targets of miRNAs that changed after exercise to determine overlap between those targets and any exercise-induced mRNA changes. Only GIMAP8 and NRF4A3 mRNAs, predicted targets of hsa-miR-10a-5p, also were exercise-responsive (S5 Table).

**Table 2:** miRNA species changing significantly 30 minutes after exercise.

Mature miRNA	Precursor	Basal	Post Exercise	Log_2_(fold increase)
hsa-miR-10a-5p	hsa-mir-10a	807 ± 120	1056 ± 134	0.408 ± 0.101
hsa-miR-30a-5p	hsa-mir-30a	3548 ± 456	5155 ± 327	0.570 ± 0.102
hsa-miR-30d-5p	hsa-mir-30d	4431 ± 454	6425 ± 595	0.541 ± 0.127
hsa-miR-22-3p	hsa-mir-22	7681 ± 948	14180 ± 1839	0.888 ± 0.191
hsa-miR-128	hsa-mir-128-1	1593 ± 173	3393 ± 685	1.027 ± 0.206
hsa-miR-128	hsa-mir-128-2	905 ± 101	1941 ± 410	1.022 ± 0.235
hsa-miR-378a-3p	hsa-mir-378a	24843 ± 3181	40779 ± 7614	0.688 ± 0.169
hsa-miR-378f	hsa-mir-378f	148 ± 24	244 ± 39	0.726 ± 0.162
hsa-miR-378a-5p	hsa-mir-378a	361 ± 36	528 ± 36	0.561 ± 0.145
hsa-miR-378g	hsa-mir-378g	75 ± 16	117 ± 32	0.611 ± 0.160
hsa-miR-378i	hsa-mir-378i	1550 ± 230	2690 ± 542	0.768 ± 0.210
hsa-miR-422a	hsa-mir-422a	74 ± 16	124 ± 36	0.689 ± 0.163
hsa-miR-532-5p	hsa-mir-532	298 ± 59	682 ± 152	1.160 ± 0.339

Data are given as Means ± SEM, units are number of specific reads normalized to total reads for a given sample. All changes shown for log2(fold stimulation) are P < 0.0004 or less (Bonferroni correction to keep family-wise error rate at 0.05).

## Discussion

A number of studies have examined how acute exercise alters gene expression in skeletal muscle [9, 10, 13]. A recent global analysis showed that acute endurance exercise conducted by healthy, middle-aged men has profound effects on gene expression in exercising muscle [1]. Among the genes most profoundly increased in that study were NR4A family members, ADAMST1 (involved in extracellular matrix metabolism), and additional transcription factors including FOS, JUNB, and EGR1 [1]. We confirmed here the previously reported exercise response for many of those genes. However, we had shown previously that the period immediately following an exercise bout is critical for defining the exercise response particularly in relationship to insulin action [6], and we proposed that lower gene expression responses to exercise in individuals with insulin sensitivity on the lower end of the normal range might exacerbate a poor response to insulin. Therefore, the primary purpose of this study was to extend and enhance previous studies by determining how exercise-induced gene expression is influenced by insulin sensitivity in a global, unbiased manner.

To accomplish this, we performed microarray analysis of RNA isolated from muscle biopsies taken at rest and 30 minutes after completion of exercise in 14 healthy subjects who also had euglycemic clamps to measure insulin sensitivity. This analysis revealed that, at the probe level, 215 mRNAs were altered significantly (Bonferroni corrected P < 0.0000015) by exercise. The vast majority of these mRNAs (198) were increased following exercise. Genes encoding transcription factor or DNA binding proteins were the most represented (about 25%). Also represented were chemokines, cell cycle regulators, genes involved in angiogenesis, and extracellular matrix formation. Genes coding cell-signaling proteins also were represented, with the beta 2 adrenergic receptor and a proton-sensing G-protein coupled receptor (GPR4) increasing, along with mRNA for an orphan receptor, GPR157. Changes in mRNA of representative genes were in general confirmed by Q-rt-PCR, as we noted previously [30]. Pathway and GO term analysis using DAVID (http://david.niaid.nih.gov) confirmed that transcriptional regulation and DNA binding GO terms were highly enriched. The MAP kinase pathway also was highly enriched, again reinforcing the notion that the immediate post-exercise period is characterized in healthy muscle by a broad response of transcription factors, stress response, growth and differentiation genes, and angiogenic factors. These early exercise-induced changes are likely to coordinate a longer term pattern of response. In this respect, the present findings confirm earlier results [1].

Because of the preponderance of transcription factors in exercise-responsive genes, we further extended previous findings [1] and our earlier results [6] by analyzing the 5’-UTRs of all exercise responsive genes to determine whether there might be a pattern of common exercise-enhanced transcription factors that could be candidates for “master” regulators of the immediate post-exercise response. One of the more intriguing transcription factors that had a binding motif significantly enriched in exercise-responsive genes was EGR1, or early growth response 1 [31]. EGR1 itself had an 11.4-fold increase in expression after exercise. EGR1 is expressed in response to a variety of stimuli [32] and is induced in electrically-contracted mouse muscle within 30 minutes [33]. Other studies show that EGR1 is responsive to MAP kinase signaling [34], which is interesting in light of the effect of exercise on MAP kinase pathway members. EGR1 also is involved in VEGF signaling [35]. These analyses, taken together with our earlier data and previous findings, suggest the possibility that EGR1, through MAP kinase and VEGF signaling pathways, may integrate the early gene expression response to exercise.

However, the primary purpose of this study was to determine the relationship between insulin sensitivity in muscle and the gene expression response to exercise. It is commonly accepted that otherwise healthy individuals display a wide range of insulin action in muscle [2]. We and others reported previously that in healthy people muscle that has lower insulin sensitivity also exhibits characteristics of “exercise resistance”, in that there are differences in gene expression responses to exercise that might subsequently contribute to even lower insulin sensitivity or altered mitochondrial function [6, 7, 11, 36, 37]. To address this question we correlated exercise-induced changes in mRNA expression at the probe level with insulin sensitivity determined using a euglycemic clamp. To minimize false positives, for this analysis we only used probes that were changed at the Bonferroni significant level of 0.00000115. Among the genes that had Bonferroni-significant exercise-induced changes that were correlated with insulin sensitivity were transcription factors such as MYC and ATF3. These transcription factors are common to many genes, and therefore if their expression after exercise is dependent upon the level of insulin sensitivity, even in healthy individuals, this suggests that there could be a widespread effect of insulin sensitivity on the exercise response. Since these effects were seen merely 30 minutes after exercise, they likely are mediating differences at later time points, compelling a thorough, global time course analysis of gene expression after exercise that is beyond the scope of the present study. Another gene, SNF1LK, also had changes that were correlated with insulin sensitivity. This genes is involved in muscle development and differentiation. SNF1LK is a CREB dependent gene that phosphorylates HDAC4 and HDAC5, which promotes expression of MEF2 isoforms and their downstream genes that mediate growth and differentiation in skeletal muscle [38]. Therefore, key exercise-induced processes involved in muscle gene expression, growth, and differentiation appear to be affected by the level of insulin sensitivity in healthy individuals, with those on the lower end of the insulin action spectrum having lower responses. These findings reinforce and extend previous studies showing that responses to exercise are related to insulin sensitivity. Blunted responses at the lower end of the insulin action spectrum that have been observed include effects on whole body or leg glucose uptake [36], mitochondrial response [37], and PGC-1α and downstream genes [6, 7, 37]. These findings lead to the working hypothesis that lower insulin sensitivity leads to lower exercise-induced gene expression response and less muscle adaptation to exercise, which could in turn lead to lower insulin action.

A second reason for performing this study was to characterize the microRNA response to exercise in healthy people in an unbiased manner by using sequencing techniques. As was the case for mRNA changes, a number of miRNAs responded briskly within 30 minutes of the end of exercise. Among these, members of the miR-30, miR-128, and miR-378 families were well represented and abundant in human muscle. Next generation sequencing techniques allowed for high specificity of quantification. The known and predicted targets of these miRNAs include several mRNAs that directly pertain to both exercise-induced mRNA responses and muscle insulin sensitivity. For example, there are multiple predicted miR-378 and miR-30 sites in the 3’UTR of the PPARA gene. Also pertinent may be that miR-378 and miR-30 have interaction sites in the 3’-UTR regions of both PPARGC1A and PPARGC1B. Because miR-378a is located within intron 1 of the PPARGC1B gene, these data support the idea that there may be a regulatory network consisting of PPARs, PGC-1 isoforms, and micro RNAs that participate in the exercise-induced gene expression response. It is conceivable that dysregulation of expression of miRNA-378 could be a component of insulin resistance in skeletal muscle and might be responsible for the decreased PGC-1α response seen after exercise in insulin resistant skeletal muscle, possibly by a feedback mechanism [6]. Recently, miR-378 isoforms have been implicated in the control of mitochondrial function and energy balance in mice [39]. Carrer and colleagues used a mouse that had miR-378-3p and -5p deleted, while leaving the PPARGC1B gene intact [39]. These mice were resistant to diet-induced obesity, possibly due to effects in liver. Thus, the changes seen in miR-378 in muscle following exercise could also have effects on mitochondrial function, metabolism or insulin sensitivity. Further studies are required to define these effects. We also searched predicted mRNA targets for miRNAs that were responsive to exercise. Of these, only hsa-miR-10a-5p and predicted mRNA targets GIMAP8 and NRF4A3 stood out as being involved in exercise responses. GIMAP8 expression was one of the few mRNAs that decreased after exercise (along with other GIMAP isoforms), and so it may be significant that hsa-miR-10a-5p expression that increased. However, the changes in GIMAP expression with respect to either exercise or skeletal muscle itself are unclear, as these genes do not have a described function in muscle. Finally, since miRNA responses were not related to insulin sensitivity, it can be concluded that alterations in these responses are not involved in the phenomenon of “exercise resistance” [6]. Nevertheless, it is becoming more evident that miRNA expression plays a role in muscle plasticity in response to aging or exercise [17, 18].

Several other studies in addition to that by Catoire *et al*. [1] have examined gene expression or protein abundance responses to an exercise bout, but have not addressed insulin sensitivity-related differences. Mahoney and colleagues studied mRNA changes in muscle from young, healthy subjects 3 after an hour of high intensity and found a number of transcription factors and genes coding mitochondrial proteins to be increased [9]. Using rats that exercised for 2 hours, McKenzie *et al*. found mRNA for a number of transcription factors and signal transduction proteins to be increased one hour after exercise, including ATF3, agreeing with the current results [10]. Jozsi *et al*. compared the response to a single bout of resistance exercise in young and healthy older men [8]. In general, the older men had greatly reduced gene expression responses, with the transcription factor EGR-1 showing an increase in older subjects and a decrease in the young men, while other genes involved in angiogenesis (VEGF) or c-jun, increased similarly. Using mice, Safdar and colleagues examined the expression of mRNAs for selected genes, including PGC-1α, after a single bout of exercise [13]. As others have shown in rodents and humans, PGC-1α mRNA increased after exercise [6, 40]. A number of other investigators have examined how exercise training, as opposed to a single bout of exercise, alters gene expression or protein abundance in skeletal muscle. These studies have used designs comparing trained and sedentary individuals, or comparing the same individuals before and after training. In general, the studies that use training [12, 41] show more consistent increases in genes coding for mitochondrial proteins or proteins involved in metabolism than were found in the current study.

In conclusion, a single bout of exercise robustly alters skeletal muscle gene expression in a manner that is related to insulin sensitivity. The response of transcription factor gene expression to exercise, especially those transcription factors that have insulin sensitivity-related responses provides avenues of research that may help to explain this phenomenon and its relevance. This study in humans may indicate the importance of the altered exercise response in insulin resistance, and point to MAP kinase and VEGF signaling, possibly through EGR1 and a limited number of transcription factors.

## Supporting Information

S1 Fig RNA was isolated as described in the text, and exercise-induced fold changes are shown for Q-rt-PCR results in relationship to microarray results. Data are Mean ± SEM (n = 6).
(EPS)

S1 Table Primers used for quantitative PCR analysis. (DOCX)

S2 Table Probe level analysis of gene expression changes following exercise. Probes shown were significantly altered by exercise at a Bonferroni-corrected P < 0. 00000115; shown are P values that were lower than the Bonferroni threshold. All data are normalized and log2 transformed and are given cumulatively as mean ± STDERR, standard error. Data for each individual also are shown (basal = pre exercise value; 30min = value 30 minutes after completion of exercise).
(XLSX)

S3 Table DAVID analysis of probes that significantly changed after exercise in all subjects. Genes were categorized into significantly enriched pathways as described in the Methods.
(DOCX)

S4 Table Enrichment of transcription factor binding sites in 5’ UTRs of genes altered in expression by acute exercise. Bonferroni P values refer to enrichment of transcription factors relative to random selection of 5’ UTRs in genome (PScan). *P < 0.05, correlation between insulin stimulated glucose disposal and exercise-induced change in gene expression.
(DOCX)

S5 Table Predicted mRNA targets of microRNAs that were changed after exercise. Diana Tools was used to predict targets (see Methods). Also shown are predicted mRNA targets that were changed significantly after exercise.
(DOCX)
