# Supplementary material for: Gene and MicroRNA Expression Responses to Exercise; Relationship with Insulin Sensitivity
Source: PLoS One. 2015 May 18;10(5):e0127089. doi: 10.1371/journal.pone.0127089 (PMC4436215; doi:10.1371/journal.pone.0127089)
Supplement: S1 Table — (DOCX) [file pone.0127089.s002.docx]

| Gene | **Forward** | **Reverse** |
| --- | --- | --- |
| EGR | ATGCAGCTGATGTCCCCGCT | AAAGACTCTGCGGTCAGGTGCT |
| FOS | AGCATGGGCTCGCCTGTCAA | TCCTGCCAATGCTCTGCGCT |
| MYC | AACGACAGCAGCTCGCCCAA | AGGAGGTTTGCTGTGGCCTCCA |
| JUNB | TGTGCGCGCAGCCCAAACTA | TCGGCCAGGTTCAGGGTCAT |
| GPCR157 | AGATCGCCTGCTTTGGGCCT | TGCCGTGTGCGCTCTGTTGA |
| GAPDH | AACCTGCCAAATATGATGAC | TCATACCAGGAAATGAGCTT |
| Cyclophilin b | GCCATGGAGCGC TTGG | CCACAGTCAGCAATGGTGATC |
| Beta actin | AAACTGGAACGGTGAAGGTG | AGAGAAGTGGGGTGGCTTTT |

S1 Table. Primers used for quantitative PCR analysis
