# Supplementary material for: Gene and MicroRNA Expression Responses to Exercise; Relationship with Insulin Sensitivity
Source: PLoS One. 2015 May 18;10(5):e0127089. doi: 10.1371/journal.pone.0127089 (PMC4436215; doi:10.1371/journal.pone.0127089)
Supplement: S3 Table — Genes were categorized into significantly enriched pathways as described in the Methods. (DOCX) [file pone.0127089.s004.docx]

S3 Table. DAVID analysis of probes that significantly changed after exercise in all subjects

| *Term* | *Genes* | | | *Enrichment* | | | *FDR* | |
| --- | --- | --- | --- | --- | --- | --- | --- | --- |
| hsa04010:MAPK signaling pathway | | HSPA1L, DUSP5, DUSP1, PDGFB, NR4A1, GADD45B, MYC, FLNB | | | | 4.0 | 2.7 | |
| hsa04350:TGF-beta signaling pathway | | ID2, ID1, SMAD7, ID3, MYC | | | | 7.7 | 3.4 | |
| GO:0030528~transcription regulator activity | | FOSL2, ELF4, TRIB3, HEY1, BCL3, ABRA, MYC, SERTAD1, EGR1, KLF6, MAFF, ERF, EGR3, EGR2, SMAD7, NR4A2, PPP1R10, NR4A1, NR4A3, JUNB, HES1, ATF3, MSX1, ID2, ID1, HEYL, MNT, BCL6B, ID3, KLF2, KLF4, DNAJB6 | | | | 3.2 | 2.06E-06 | |
| GO:0003700~transcription factor activity | | EGR1, ERF, MAFF, KLF6, EGR3, EGR2, FOSL2, ELF4, SMAD7, NR4A2, NR4A1, NR4A3, JUNB, ATF3, MSX1, HEY1, HEYL, MNT, BCL3, KLF2, MYC, KLF4 | | | | 3.4 | 8.58E-04 | |
| GO:0016564~transcription repressor activity | | ERF, TRIB3, JUNB, HES1, MSX1, ATF3, ID2, ID1, MNT, BCL6B, ID3, DNAJB6, KLF4 | | | | 6.3 | 0.001 | |
| GO:0016563~transcription activator activity | | EGR1, KLF6, ELF4, NR4A2, MNT, NR4A1, ABRA, NR4A3, KLF2, SERTAD1, KLF4, JUNB | | | | 4.5 | 0.08 | |
| GO:0043565~sequence-specific DNA binding | | EGR1, ERF, MAFF, FOSL2, SMAD7, ELF4, NR4A2, NR4A1, NR4A3, JUNB, MSX1, ATF3, MYC, KLF4 | | | | 3.5 | 0.17 | |
| GO:0005525~GTP binding | | GIMAP4, GIMAP6, GIMAP7, GIMAP8, TUBB6, RIT1, RASD1, TUBB4, GBP1, GIMAP1 | | | | 4.1 | 0.83 | |
| GO:0019001~guanyl nucleotide binding | | GIMAP4, GIMAP6, GIMAP7, GIMAP8, TUBB6, RIT1, RASD1, TUBB4, GBP1, GIMAP1 | | | | 3.99 | 0.99 | |
| GO:0032561~guanyl ribonucleotide binding | | GIMAP4, GIMAP6, GIMAP7, GIMAP8, TUBB6, RIT1, RASD1, TUBB4, GBP1, GIMAP1 | | | | 3.99 | 0.99 | |
| GO:0003677~DNA binding | | FOSL2, ELF4, HEY1, BCL3, SOX17, MYC, EGR1, KLF6, MAFF, ERF, EGR3, EGR2, SMAD7, NR4A2, PPP1R10, NR4A1, NR4A3, SNAI1, JUNB, HES1, ATF3, MSX1, HEYL, MNT, BCL6B, KLF2, KLF4, DNAJB6 | | | | 1.8 | 1.5 | |
| GO:0003714~transcription corepressor activity | | ERF, ATF3, MNT, TRIB3, ID3, JUNB | | | | 6.3 | 3.1 | |
| GO:0003690~double-stranded DNA binding | | EGR1, KLF6, MYC, KLF4, JUNB | | | | 7.9 | 4.5 | |
|  | |  |  | |  | | |  |

Probe level analysis of gene expression changes following exercise. Probes shown were significantly altered by exercise at a Bonferroni-corrected P < 0. 00000115; shown are P values that were lower than the Bonferroni threshold. All data are normalized and log2 transformed and are given cumulatively as mean ± STDERR, standard error. Data for each individual also are shown (basal = pre exercise value; 30min = value 30 minutes after completion of exercise).
