# Supplementary material for: Gene and MicroRNA Expression Responses to Exercise; Relationship with Insulin Sensitivity
Source: PLoS One. 2015 May 18;10(5):e0127089. doi: 10.1371/journal.pone.0127089 (PMC4436215; doi:10.1371/journal.pone.0127089)
Supplement: S4 Table — Bonferroni P values refer to enrichment of transcription factors relative to random selection of 5’ UTRs in genome (PScan). *P < 0.05, correlation between insulin stimulated glucose disposal and exercise-induced change in gene expression. (DOCX) [file pone.0127089.s005.docx]

S4 Table. Enrichment of transcription factor binding sites in 5’ UTRs of genes altered in expression by acute exercise.

| Transcription  Factor | Bonferroni P | 5’ - Motif - 3’ | R with insulin sensitivity |
| --- | --- | --- | --- |
| SP1 | 0.0000000651 | GGGCGGG | 0.47 |
| NFKB1 | 0.0000914 | GGGAATTTCC | 0.58* |
| RELA | 0.042 | GGGAATTTCC | 0.67* |
| AP2 | 0.000178 | GCCNNNGGC | 0.22 |
| EGR1 | 0.0237 | CGCCCCCGC | 0.13 |
| Bonferroni P values refer to enrichment of transcription factors relative to random selection of 5’ UTRs in genome (PScan). *P < 0.05, correlation between insulin stimulated glucose disposal and exercise-induced change in gene expression. | | | |
