# Supplementary material for: Gene and MicroRNA Expression Responses to Exercise; Relationship with Insulin Sensitivity
Source: PLoS One. 2015 May 18;10(5):e0127089. doi: 10.1371/journal.pone.0127089 (PMC4436215; doi:10.1371/journal.pone.0127089)
Supplement: S5 Table — Diana Tools was used to predict targets (see Methods). Also shown are predicted mRNA targets that were changed significantly after exercise. (DOCX) [file pone.0127089.s006.docx]

S5 Table. Predicted targets of miRNAs significantly changed by exercise.

| Mature miRNA | Predicted Targets | Predicted Targets Among mRNAs Changed by Exercise |
| --- | --- | --- |
| hsa-miR-10a-5p | <http://diana.imis.athena-innovation.gr/DianaTools/index.php?r=microT_CDS/results&keywords=hsa-miR-10a-5p&genes=&mirnas=hsa-miR-10a-5p%20&descr=&threshold=0.7> | GIMAP8, NR4A3 |
| hsa-miR-30a-5p | <http://diana.imis.athena-innovation.gr/DianaTools/index.php?r=microT_CDS/results&keywords=hsa-miR-30a-5p&genes=&mirnas=hsa-miR-30a-5p%20&descr=&threshold=0.7> |  |
| hsa-miR-30d-5p | <http://diana.imis.athena-innovation.gr/DianaTools/index.php?r=microT_CDS/results&keywords=hsa-miR-30d-5p&genes=&mirnas=hsa-miR-30d-5p%20&descr=&threshold=0.7> |  |
| hsa-miR-22-3p | <http://diana.imis.athena-innovation.gr/DianaTools/index.php?r=microT_CDS/results&keywords=hsa-miR-22-3p&genes=&mirnas=hsa-miR-22-3p%20&descr=&threshold=0.7> |  |
| hsa-miR-128 | <http://diana.imis.athena-innovation.gr/DianaTools/index.php?r=microT_CDS/results&keywords=hsa-miR-128&genes=&mirnas=hsa-miR-128%20&descr=&threshold=0.7> |  |
| hsa-miR-378a-3p | <http://diana.imis.athena-innovation.gr/DianaTools/index.php?r=microT_CDS/results&keywords=hsa-miR-378a-3p&genes=&mirnas=hsa-miR-378a-3p%20&descr=&threshold=0.7> |  |
| hsa-miR-378a-5p | <http://diana.imis.athena-innovation.gr/DianaTools/index.php?r=microT_CDS/results&keywords=hsa-miR-378a-5p&genes=&mirnas=hsa-miR-378a-5p%20&descr=&threshold=0.7> |  |
| hsa-miR-378f | <http://diana.imis.athena-innovation.gr/DianaTools/index.php?r=microT_CDS/results&keywords=hsa-miR-378f&genes=&mirnas=hsa-miR-378f%20&descr=&threshold=0.7> |  |
| hsa-miR-378g | <http://diana.imis.athena-innovation.gr/DianaTools/index.php?r=microT_CDS/results&keywords=hsa-miR-378g&genes=&mirnas=hsa-miR-378g%20&descr=&threshold=0.7> |  |
| hsa-miR-378i | <http://diana.imis.athena-innovation.gr/DianaTools/index.php?r=microT_CDS/results&keywords=hsa-miR-378i&genes=&mirnas=hsa-miR-378i%20&descr=&threshold=0.7> |  |
| hsa-miR-422a | <http://diana.cslab.ece.ntua.gr/DianaToolsNew/index.php?r=tarbase/index&mirnas=MIMAT0001339> |  |
| hsa-miR-532-5p | <http://diana.cslab.ece.ntua.gr/DianaToolsNew/index.php?r=tarbase/index&mirnas=MIMAT0002888> |  |

Links are given to predicted miRNA targets determined using the microT-CDS tool available on Diana Tools (<http://diana.imis.athena-innovation.gr/DianaTools/index.php?r=site/index>). Also shown are mRNAs for genes that were significantly altered by exercise that also appeared among the most significant 30 predicted miRNA targets.
